# Early graft calcification without graft dysfunction after living donor liver transplantation: two case reports

**DOI:** 10.1007/s12328-021-01471-x

**Published:** 2021-08-22

**Authors:** Peilin Li, Masaaki Hidaka, Yu Huang, Takanobu Hara, Kantoku Nagakawa, Hajime Matsushima, Takayuki Tanaka, Tomohiko Adachi, Akihiko Soyama, Weili Gu, Kengo Kanetaka, Susumu Eguchi

**Affiliations:** 1 Department of Surgery, Nagasaki University Graduate School of Biomedical Sciences, 1-7-1 Sakamoto, Nagasaki, Nagasaki 852-8102 Japan; 2 Department of Surgery, Guangzhou First People’s Hospital, School of Medicine, South China University of Technology, Guangzhou, Guangdong China

**Keywords:** Living donor liver transplantation, Graft calcification, Ischemia–reperfusion injury

## Abstract

Graft calcification after liver transplantation (LT) has seldom been reported, but almost of all previously reported cases have been attributed to graft dysfunction. We herein report two cases of graft calcification without liver dysfunction after living donor liver transplantation (LDLT). Two patients who underwent LDLT were found to have graft calcification in the early postoperative period (< 1 month). Calcification in the first case was found at the cut edge of the liver at post-operative day (POD) 10, showing a time-dependent increase in calcification severity. The second patient underwent hepatic artery re-anastomosis due to hepatic artery thrombosis on POD4 and received balloon-occluded retrograde transvenous obliteration of the splenic kidney shunt due to decreased portal vein blood flow on POD6. She was found to have diffuse hepatic calcification in the distant hepatic artery area at 1-month post-operation followed by gradual graft calcification at the resection margin at 6-month post-operation. Neither case showed post-operative graft dysfunction. Calcification of the liver graft after LDLT is likely rare, and graft calcification does not seem to affect the short-term liver function in LDLT cases. We recommend strictly controlling the warm/cold ischemia time and reducing the physical damage to the donor specimen as well as monitoring for early calcification by computed tomography.

## Introduction

Liver transplantation (LT) is a treatment option for end-stage liver disease and acute liver failure. Various injuries, such as inflammation and primary benign or malignant tumor, have been reported to cause pathological liver calcification [1, 2]. Hepatic calcification reportedly develops in the degenerative area of the hepatic lobule following ischemia after serious shock lasting for 2 days [2]. Diffuse hepatocellular calcification has also been reported in patients receiving chronic hemodialysis after ischemic hepatitis [3]. Graft calcification after LT has seldom been reported, but almost of all previously reported cases of graft calcification after LT were attributed to graft dysfunction [4–6].

We herein report two cases with graft calcification without liver dysfunction after living donor liver transplantation (LDLT).

## Case report

### Case 1

A 50-year-old woman, diagnosed with Caroli’s disease, was performed underwent LDLT in 2009. She had a medical history of chronic renal failure without dialysis. Two years later, she underwent re-transplantation because of liver dysfunction caused by chronic anti-graft rejection. The living donor graft was transplanted with a blood-compatible extended left graft lobe. The graft weight/standard liver volume (GW/SLV) was 35.7%. The total operation time was 674 min, including 124 min of ischemia time. The blood loss was 5800 g. She had a normal liver function post re-transplantation.

**Fig. 1:**
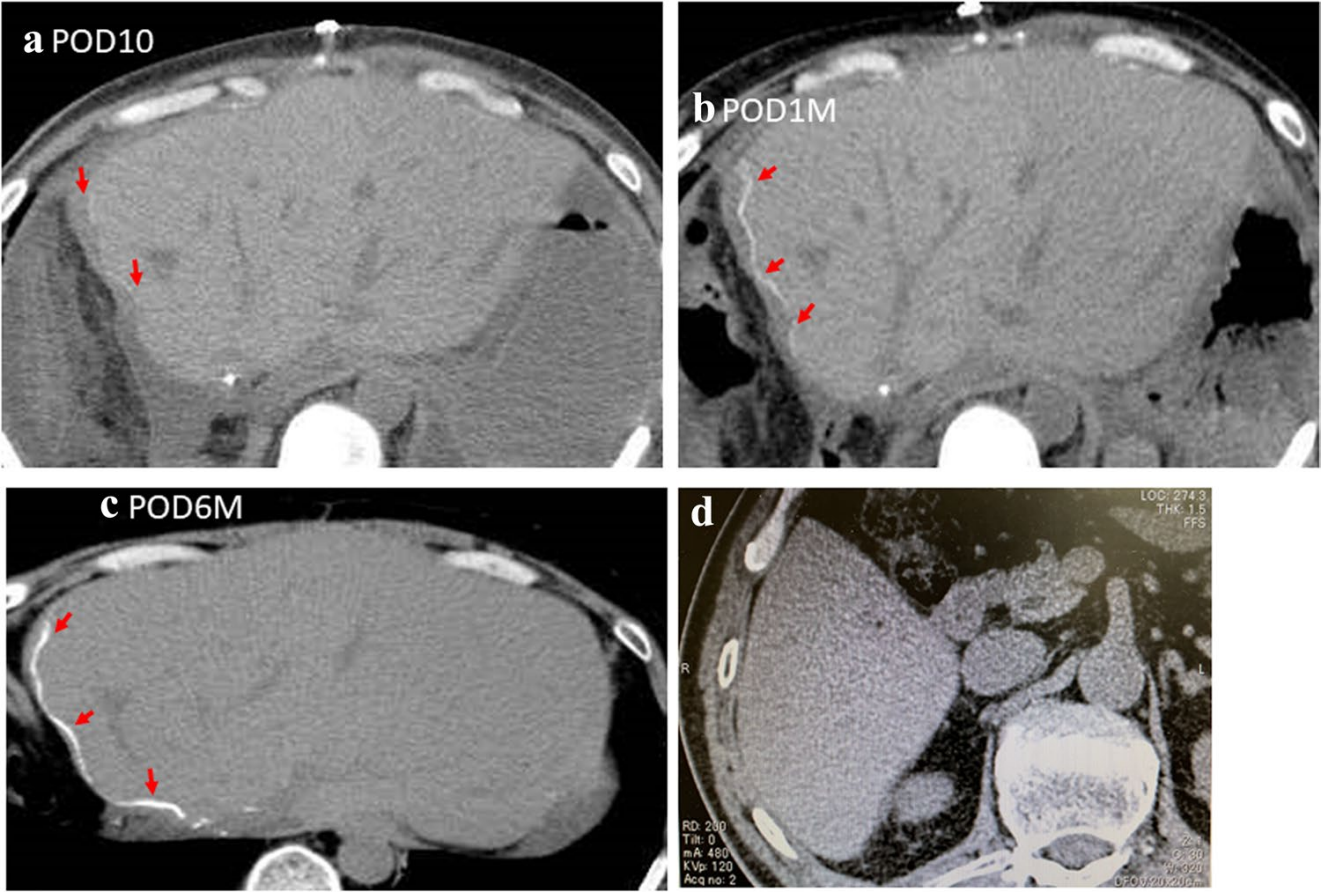
CT image from case 1 showing the accumulation of calcium in the surrounding tissues of the wound. **a** POD10, the surrounding tissue of the wound showed slight calcification. **b** One month after LDLT, the calcification had increased. **c** Six months after LDLT, the graft calcification had further increased. **d** Postoperative CT image showed on presence of calcification in the donor’s liver

On POD10, computed tomography (CT) showed slight calcification in the transection area of the liver (Fig. 1a). One month after re-transplantation, CT showed obvious calcification distributed along the cut edge (Fig. 1b). Calcification was found to have further increased at 6 months later (Fig. 1c). Serum calcium level was checked after LDLT. The serum calcium level was 1.14 – 1.27 mmol/L and the blood phosphorus was 3.0 mg/dL. During this course, she had a roughly normal liver function, and she had the liver function of TP 54 g/L, ALB 31 g/L, ASL/ALT 13/13 U/L and ALP 40–110 U after 6 months. The patient ultimately died of recurrence of occult cholangiocarcinoma at 24 months after re-transplantation. Postoperative CT examination did not find the presence of calcification in the donor’s liver (Fig. 1d).

### Case 2

A 40-year-old woman, diagnosed with hepatitis C virus-related liver cirrhosis, underwent LDLT with a blood-incompatible extended left graft lobe in 2013. She had no other remarkable medical history. The GW/SLV was 39.3%. The total operation time was 886 min, including 170 min of ischemia time. The total blood loss was 3300 g.

**Fig. 2:**
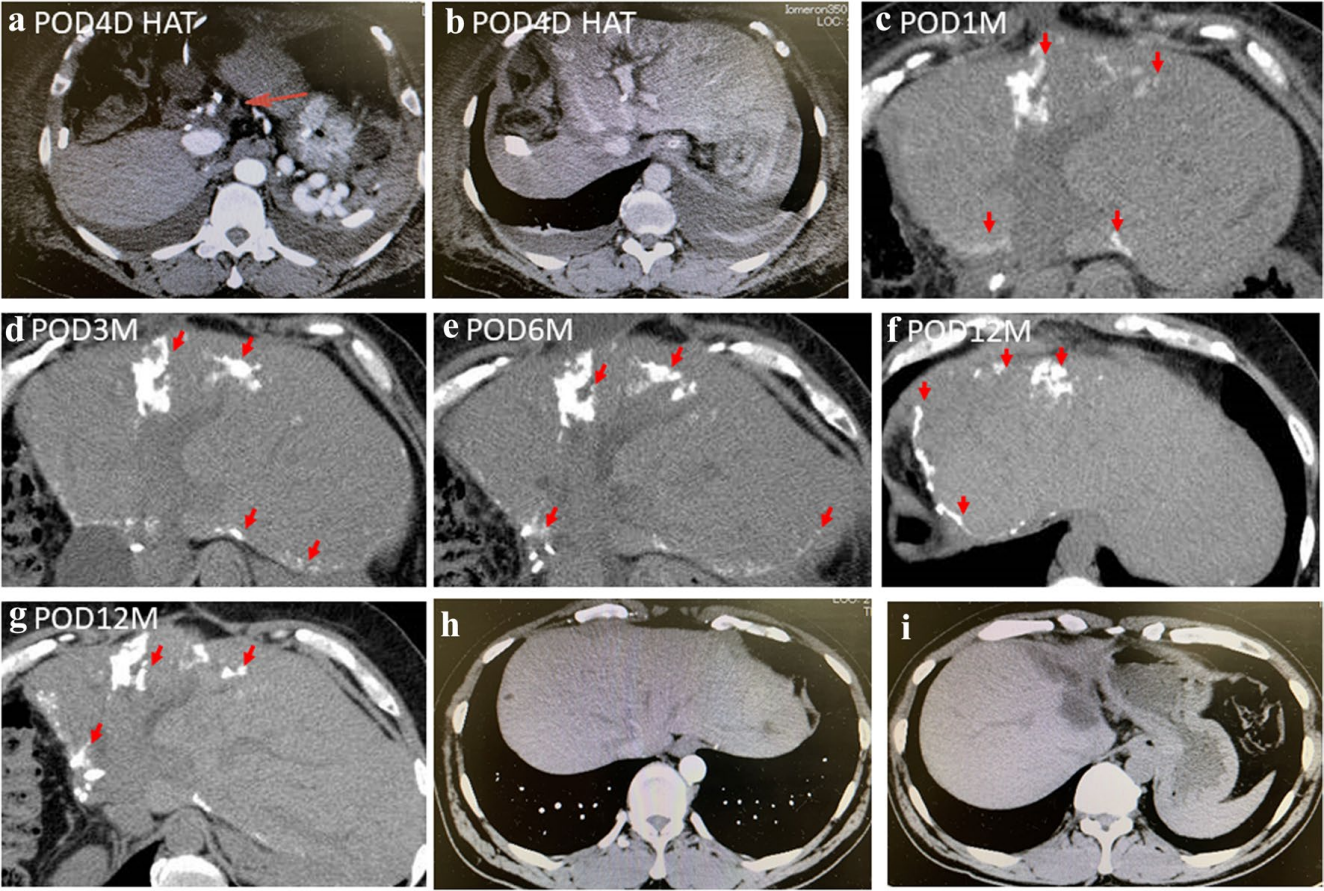
CT image from case 2 showing the accumulation of calcium in the wide range of the liver. **a**, **b** CT images showed the hepatic artery thrombosis on POD4. **c** One month after LDLT, extensive calcification of the liver graft was found during the reexamination. **d** Three months and **e** 6 months after LDLT, the area of calcification in the liver had increased compared to before. **f**, **g** CT images showed extensive graft calcification 1 year after LDLT. **h**, **i** Preoperative and postoperative images showed no presence of liver calcification in the donor liver

On POD4, she underwent hepatic artery re-anastomosis due to hepatic artery thrombosis (HAT) (Fig. 2a, b). On POD6, balloon-occluded retrograde transvenous obliteration was performed on the splenic kidney shunt due to a decreased portal vein blood flow. CT on POD30 showed diffuse hepatic calcification in the distant hepatic artery area (Fig. 2c). CT performed at 3 and 6 months of follow-up showed a gradual increase in the hepatic calcification area (Fig. 2d, e). The linear calcifications at the resection margin were also found to have gradually increased over time (Fig. 2f, g). During this period, the serum calcium level as well as hormones that was associated with calcium metabolism were checked. The serum calcium level was 1.16 – 1.26 mmol/L, the blood phosphorus was 4.3 mg/dL and the preoperative parathyroid hormone (PTH) was 21.8 pg/ml. Two months after the operation before discharge, her liver function tests were AST/ALT 93/63 U/L and ALP 1226 U. At the 1-year postoperative re-examination, the liver function were AST/ALT 64/54 U/L and ALP 380 U. The patient is still alive and maintain a good condition by taking antiviral agents without liver dysfunction in the outpatient clinic until now. Preoperative (Fig. 2h) CT examination of the donor’s liver did not find the presence of calcification in the donor’s liver, nor did it find the presence of liver calcification at postoperative (Fig. 2i) re-examination.

## Discussion

Graft calcification after LT is underreported with a rare rate. As of December 2020 in our department, we had performed 319 cases of LT, including 18 cases of deceased donor liver transplantation (DDLT). We experienced these two cases with graft calcification in early period (within 1 month) after LDLT without graft dysfunction. There might actually be more cases needed retrospective study.

We reviewed the previously reported cases of graft calcification after LT (Table 1). Tzimas et al. reported two patients who underwent DDLT and developed graft calcification at the early post-transplant period [6]. Both patients with clinical and biochemical evidence of primary graft dysfunction demonstrated calcification on light and electron microscopy. In addition, one patient had macroscopic evidence of calcification on cross-sectional imaging. Subsequently, Talmon et al. reported one case of dystrophic calcification within hepatocytes occurring in a liver allograft affected by severe ischemia–reperfusion (IR). Biochemical staining revealed that the mineralized material was calcium phosphate (likely hydroxyapatite) [5]. On electron microscopy, the hepatocyte cytoplasm was found to be filled with variably calcified vacuoles, a subset of which likely represented swollen mitochondria. Jeng et al. later reported a case of calcification after LDLT. The calcification was noted in the ischemic areas of the liver graft, probably resulting from congestion necrosis after 8 weeks [4]. All four of those reported cases underwent hemodynamic changes related to IR injury, which resulted in liver dysfunction.

**Table 1:** Reported cases of hepatic calcification after LT

Refs	patient’s info	Disease	Type of LT	Initial point of graft calcification	Possible causes	Outcome
Tzimas, BMC Surgery [6]	65 years, Man	HBV–cirrhosis	DDLT	POD2	Patent HA/PV/HV severe reperfusion injury	Primary graft dysfunction. Died on POD12
Tzimas, BMC Surgery [6]	55 years, man	HBV–cirrhosis	DDLT	POD3	Patent HA/PV/HV	Died for ventricular fibrillation after re-transplantation
Talmon. Ultrastructural Pathology [5]	65 years, woman	NASH–cirrhosis	DDLT	POD3	Severe IR	Died from cardiac arrest after re-transplantation
Jeng. Annals of hepatology [4]	46 years, woman	HBV–cirrhosis, HCC	LDLT	8th week	Torsion of the hepatic vein	Died of pneumonia and sepsis at 13th week post-operation
Present report	50 years, woman	Caroli disease ACR for re-transplantation	LDLT	4th week	Unknown	Died for the recurrence of occult CCA after 24 months of re-transplantation
Present report	40 years, Woman	HCV–cirrhosis	LDLT	POD10	HAT	Maintained with antiviral agents without liver dysfunction

*LT* liver transplantation, *HBV* hepatitis B virus, *DDLT* deceased donor liver transplantation, *LDLT* living donor liver transplantation, *IR* ischemia injury, *POD* post-operation day

Based on these previously reported findings, calcification after LT may be caused by ischemic injury. This may further affect the function of the graft and even the survival of the patients. Following IR, balanced apoptosis occurs, occasionally accompanied by necrosis of hepatocytes, which translates into cell swelling, distension of various cellular organelles, clumping and random degradation of nuclear DNA, extensive plasma membrane endocytosis and autophagy [7]. In a preliminary report conducted by Tzimas et al. [8, 9], the authors investigated the correlation of cell necrosis and tissue calcification with IR injury after LT. They found that the clinically observed degree of liver dysfunction after LT correlates well with ultrastructural modifications, including calcification and vacuole formation. Therefore, the IR injury was considered an underlying mechanism of hepatic calcification, which is usually accompanied by graft dysfunction.

Ischemic stress has been previously reported to induce calcium accumulation at the cellular level by an impaired energy metabolism and/or plasmalemmal alterations. The elevated intracellular calcium concentration causes cytoskeletal modification, which alters the cell shape, as well as activation of phospholipases, resulting in perpetuation of membrane damage and, finally, mitochondrial calcification [10].

In addition, the cellular and molecular mechanisms underlying abnormal calcification following IR injury in LT have also been investigated [8, 9]. Abundant myofibroblasts were detected in regions surrounding and within the area of calcification. In these pre-calcified and calcified areas, myofibroblasts expressed bone-specific matrix proteins, such as osteopontin, type 1 collagen and bone sialoprotein. In addition, transforming growth factor beta (TGFβ)-1 and BMP (Bone morphogenetic protein)-2, two growth factors implicated in osteoblast differentiation, and Runx-2 and Msx-2, two transcription factors target of TGFβ-1 and BMP-2, were also expressed in these myofibroblasts. These data suggested that liver calcification following transplantation may be a consequence of precipitation of hydroxylapatite emanating from necrotic or apoptotic hepatocytes associated with the proliferation of myofibroblasts expressing bone-specific matrix proteins.

In the present study, both cases underwent LDLT and were found to have graft calcification along the surgical cut margins without graft dysfunction. The calcification deposited in the edge of the incision wound seemed to be the result of hepatocyte apoptosis and necrosis caused by surgical manipulation. Furthermore, in case 2, the graft calcification was more obvious in the distant hepatic artery area than surgical cut margins and might have been caused by ischemic events due to HAT and a reduced portal vein blood flow after LDLT.

As shown in Table 1, among the six reported cases, including the two cases described in our present report, three underwent DDLT, while the others underwent LDLT. The appearance of graft calcification seems to have occurred earlier in the DDLT cases than in LDLT cases. Furthermore, the rates of graft dysfunction were higher in the DDLT cases than in the LDLT cases, possibly due to the longer ischemic time in the DDLT cases. A prolonged cold ischemia time was reported to impair the graft function, increase post-transplantation complications and reduce the survival rate [11, 12].

Based on the short-term CT results (within 1 year), the severity and size of calcifications gradually increased with time. However, the long-term impact of such graft calcification on the transplanted liver remains unclear, so further long-term monitoring is necessary. The mechanism underlying the calcification occurring at the surgical incision surface is unclear, as most of the LDLT cases showed no calcification at the surgical incision area. Therefore, a retrospective investigation with LT cases should be performed in the future.

In conclusion, calcification of the liver graft after LDLT is rare. While such graft calcification was not shown to affect the short-term liver function in LDLT cases, whether or not graft calcification affects the long-term liver function is a matter of concern. We recommend strictly controlling the warm/cold ischemia time and reducing the physical damage to the donor graft while performing early monitoring of the calcification by CT.

## Data Availability

Not applicable.

## Abbreviations

LT: Liver transplantation
POD: Post-operative day
LDLT: Living donor liver transplantation
DDLT: Deceased donor liver transplantation
EGC: Early graft calcification
HAT: Hepatic artery thrombosis
PVT: Portal vein thrombosis
CT: Computed tomography
ALP: Alkaline phosphatase
ALT: Alanine aminotransferase
AST: Aspartate aminotransferase
BMP: Bone morphogenetic protein
